# Endoplasmic Reticulum Protein TXNDC5 Interacts with PRDX6 and HSPA9 to Regulate Glutathione Metabolism and Lipid Peroxidation in the Hepatic AML12 Cell Line

**DOI:** 10.3390/ijms242417131

**Published:** 2023-12-05

**Authors:** Seyed Hesamoddin Bidooki, Javier Sánchez-Marco, Roberto Martínez-Beamonte, Tania Herrero-Continente, María A. Navarro, María J. Rodríguez-Yoldi, Jesús Osada

**Affiliations:** 1 Departamento de Bioquímica y Biología Molecular y Celular, Facultad de Veterinaria, Instituto de Investigación Sanitaria de Aragón, Universidad de Zaragoza, E-50013 Zaragoza, Spain; h.bidooki94@gmail.com (S.H.B.); javiersanchezmarc@gmail.com (J.S.-M.); romartin@unizar.es (R.M.-B.); taniaherrero1992@gmail.com (T.H.-C.); angelesn@unizar.es (M.A.N.); 2 CNRS, IPREM, Universite de Pau et des Pays de l’Adour, E2S UPPA, 64 000 Pau, France; 3 MANTA—Marine Materials Research Group, Universite de Pau et des Pays de l’Adour, E2S UPPA, 64 600 Anglet, France; 4 Instituto Agroalimentario de Aragón, CITA-Universidad de Zaragoza, E-50013 Zaragoza, Spain; mjrodyol@unizar.es; 5 Centro de Investigación Biomédica en Red de Fisiopatología de la Obesidad y Nutrición (CIBEROBN), Instituto de Salud Carlos III, E-28029 Madrid, Spain; 6 Departamento de Farmacología, Fisiología, Medicina Legal y Forense, Facultad de Veterinaria, Instituto de Investigación Sanitaria de Aragón, Universidad de Zaragoza, E-50013 Zaragoza, Spain

**Keywords:** protein interaction, TXNDC5, endoplasmic reticulum, PRDX6, HSPA9, glutathione, lipid peroxidation, liver

## Abstract

Non-alcoholic fatty liver disease or steatosis is an accumulation of fat in the liver. Increased amounts of non-esterified fatty acids, calcium deficiency, or insulin resistance may disturb endoplasmic reticulum (ER) homeostasis, which leads to the abnormal accumulation of misfolded proteins, activating the unfolded protein response. The ER is the primary location site for chaperones like thioredoxin domain-containing 5 (TXNDC5). Glutathione participates in cellular oxidative stress, and its interaction with TXNDC5 in the ER may decrease the disulfide bonds of this protein. In addition, glutathione is utilized by glutathione peroxidases to inactivate oxidized lipids. To characterize proteins interacting with TXNDC5, immunoprecipitation and liquid chromatography–mass spectrometry were used. Lipid peroxidation, reduced glutathione, inducible phospholipase A_2_ (iPLA_2_) and hepatic transcriptome were assessed in the AML12 and TXNDC5-deficient AML12 cell lines. The results showed that HSPA9 and PRDX6 interact with TXNDC5 in AML12 cells. In addition, TXNDC5 deficiency reduced the protein levels of PRDX6 and HSPA9 in AML12. Moreover, lipid peroxidation, glutathione and iPLA_2_ activities were significantly decreased in TXNDC5-deficient cells, and to find the cause of the PRDX6 protein reduction, proteasome suppression revealed no considerable effect on it. Finally, hepatic transcripts connected to PRDX6 and HSPA9 indicated an increase in the *Dnaja3*, *Mfn2* and *Prdx5* and a decrease in *Npm1*, *Oplah*, *Gstp3*, *Gstm6*, *Gstt1*, *Serpina1a*, *Serpina1b*, *Serpina3m*, *Hsp90aa1* and *Rps14* mRNA levels in AML12 KO cells. In conclusion, the lipid peroxidation system and glutathione mechanism in AML12 cells may be disrupted by the absence of TXNDC5, a novel protein–protein interacting partner of PRDX6 and HSPA9.

## 1. Introduction

Non-alcoholic fatty liver disease (NAFLD) or steatosis, an accumulation of fat in the liver independently of alcohol abuse, has come to be a burgeoning hassle due to its prevalence in the standard population [1,2]. NAFLD may evolve into a variety of pathological conditions, such as steatohepatitis, cirrhosis and hepatocellular cancer [3]. In addition to impairing lipid metabolism [4,5], hepatic steatosis also causes profound changes in whole-genome expression [2].

Lipid metabolism, calcium homeostasis, protein synthesis, post-translational modification, and trafficking are all regulated by the cellular organelle known as the endoplasmic reticulum (ER) [6,7]. In fact, one-third of proteins are transported through the ER, where the correct formation of disulfide bonds depends on the oxidative environment that is kept by the glutathione balance [8,9]. Disturbed homeostasis within the ER, resulting from excessive levels of free fatty acid, depletion of calcium, or insulin resistance, can disturb ER functions and induce the pathological accumulation of misfolded proteins, commonly called the unfolded protein response (UPR) [10,11,12,13].

Thioredoxin domain-containing 5 (TXNDC5) has three thioredoxin-like domains, is a member of the nineteen existing protein disulfide isomerase (PDI) families of oxidoreductases, is predominantly expressed in the ER, and plays a critical role in signal transduction and cancer development [2,14,15]. PDIs are widely distributed proteins whose activity promotes a cysteine–disulfide–thiol exchange that reduces disulfides in other proteins. Protein stability, damage resistance, and half-lives can all be improved by PDIs, which are crucial in determining the structure and function of proteins [16,17,18]. TXNDC5 helps proteins fold appropriately by forming disulfide connections through its thioredoxin domains and is particularly abundant in liver and endothelial tissues [13]. Induced expression of TXNDC5 has been observed in a number of cancers, including of the cervix, colon, stomach, prostate, liver, and lung [19,20]. TXNDC5 has been linked to a number of biological processes, including antioxidation, promotion of angiogenesis, energy metabolism, involvement in cellular inflammation and the function of reduced glutathione [1,9]. The interaction of TXNDC5 with glutathione in the ER may reduce the disulfide bonds of this protein [21]. In addition, numerous processes use glutathione, and it is consumed by glutathione peroxidases to decrease oxidized lipids [22,23]. Recently, the absence of TXNDC5 under fasting stress led to a prodromal stage of sterile inflammation and increased lipid peroxidation. This could be due to the antioxidant characteristics of TXNDC5 and its involvement in several cellular processes [9,24]. However, the molecular mechanisms of TXNDC5 have not been well studied, including its role in liver metabolism. In this study, we found that TXNDC5 is a novel protein–protein interaction partner of PRDX6 and HSPA9, and their lack of interaction in mouse hepatocytes may contribute to the impairment of the glutathione mechanism and lipid peroxidation in the development of liver disease and its possible repercussions.

## 2. Results

### 2.1. Identification of PRDX6 and HSPA9 as Novel TXNDC5-Interacting Proteins in the AML12 Cell Line

**Figure 1:**
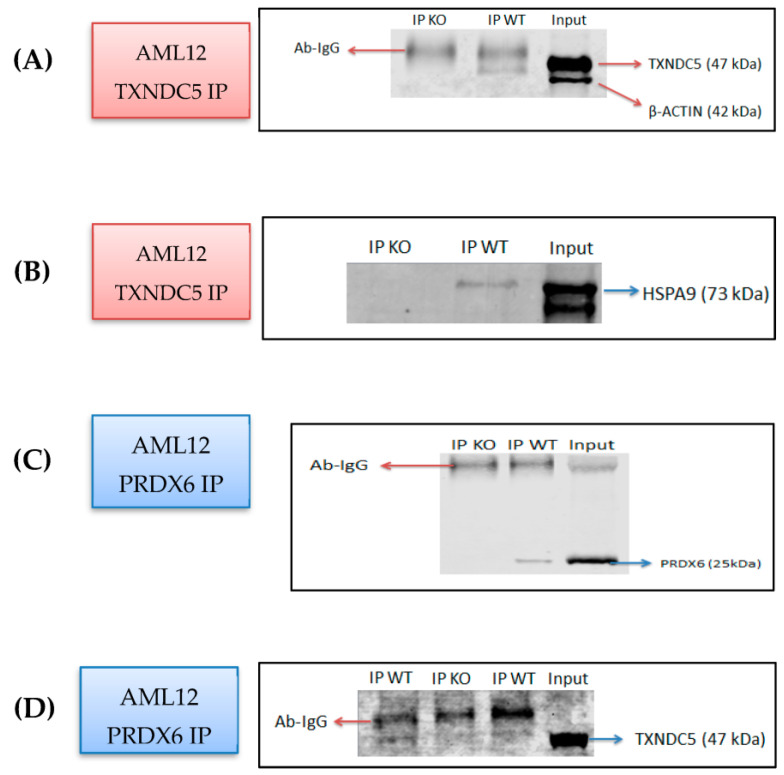
Confirmation of proteins interacting with TXNDC5 observed via Co-IP and mass spectrometry. Co-immunoprecipitation assays of AML12 WT and KO were immunoprecipitated with the TXNDC5 antibody, and the precipitates were analyzed by means of Western blot using an antibody against (**A**) TXNDC5 and (**B**) HSPA9. (**C**) AML12 WT and KO were co-immunoprecipitated with the PRDX6 antibody, and (**D**) the precipitates were examined via Western blotting with the anti-TXNDC5 antibody. Description of the lanes from left to right (**A**–**C**): first lane: co-immunoprecipitation in KO AML12 cells sample, second lane: co-immunoprecipitation in WT AML12 cells sample, third lane: WT AML12 cells sample without co-immunoprecipitation as input. (**D**) First and third lanes: co-immunoprecipitation in WT AML12 cells sample, second lane: co-immunoprecipitation in KO AML12 cells sample; fourth lane: WT AML12 cells sample without co-immunoprecipitation as input.

In the AML12 cell line, due to its relevance to hepatic functions and ER processes, a study aimed at elucidating novel interacting partners of TXNDC5, a protein involved in redox regulation and protein folding [15], is crucial for understanding the proper functioning of TXNDC5 in the ER proteostasis network and cellular processes and signaling pathways. In order to find out the putative TXNDC5 interacting proteins, co-immunoprecipitation (Co-IP) and mass spectrometry were used. In Supplementary Table S2 are listed the identified proteins in the wild-type (WT) AML12 and absent in the TXNDC5 knockout cell lines. A total of 24 proteins were differentially immunoprecipitated in the presence or absence of TXNDC5 using an anti-TXNDC5 antibody. Considering the percentage of the protein sequence covered by the peptides (coverage > 10%), the presence of the number of distinct and unique peptides, and the total number of identified peptide sequences (peptide spectrum matches, (PSMs)), two proteins were clearly identified: HSPA9 and PRDX6. These findings were confirmed using Western blot analysis (Figure 1). To validate the immunoprecipitation, Western blot detection of TXNDC5 in immunoprecipitated WT and TXNDC5-KO AML12 cells was carried out using an anti-TXNDC5 antibody (Figure 1A). As expected, the band corresponding to TXNDC5 was not observed in the TXNDC5-KO AML12 cells. When the same preparations were revealed using an anti-HSPA9 antibody, no band was observed in the TXNDC5-KO AML12 cells (Figure 1B). Using an anti-PRDX6 for immunoprecipitation (Figure 1C), no TXNDC5 band was detected in the cells lacking TXNDC5 (Figure 1D). These results confirm the interaction of TXNDC5 with HSPA9 and PRDX6 in the AML12 cell line. The identification of PRDX6 and HSPA9 as novel interacting partners of TXNDC5 in the AML12 cell line expands our understanding of the protein’s cellular context, shedding light on its potential roles in redox regulation, lipid peroxidation, and ER–mitochondrial crosstalk.

### 2.2. HSPA9 mRNA and Protein Levels Are Differentially Influenced by the Absence of TXNDC5

**Figure 2:**
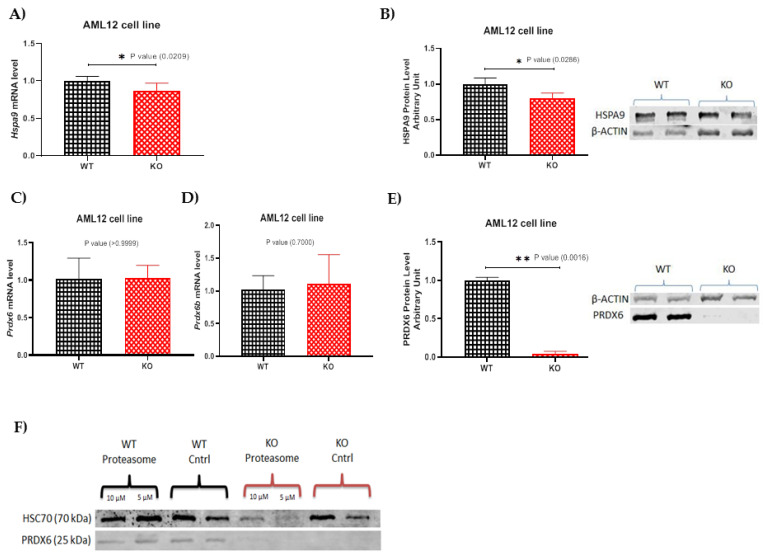
(**A**,**B**) Expression pattern of HSPA9. mRNA level of *Hspa9* in the (**A**) AML12 cell line. (**B**) Protein level of HSPA9 and its Western blot in the hepatic cell line. (**C**–**E**) Expression pattern of PRDX6. The RNA expression level of *Prdx6* (**C**) in the AML12 cell line and (**D**) the mRNA level of *Prdx6b* is shown in the AML12 cell line. (**E**) The protein level of PRDX6 was analyzed using Western blot in the hepatic cell lines. The Mann–Whitney U test for pairwise comparisons was used for statistical analysis; * *p* < 0.05, ** *p* < 0.01. (**F**) Effect of a proteasome inhibitor on the PRDX6 protein levels in the AML12 WT and KO cell lines. Cells were exposed to 5 and 10 µM MG-132 as a proteasome inhibitor for 24 h; then, the proteins were extracted and analyzed via Western blot. The MG-132-exposed WT cells displayed the same PRDX6 bands as the control group; even so, the KO cells did not show any bands. (HSC70 was used as a control protein.)

HSPA9 is a mitochondrial chaperone protein involved in protein folding and transport [25]. Its interaction with TXNDC5 suggests possible crosstalk between ER and mitochondrial protein-folding mechanisms. To understand the effect of TXNDC5 deficiency on hepatic cells, we investigated whether the HSPA9 mRNA and protein expressions were altered by the absence of TXNDC5 and the lack of its interaction. Elimination of TXNDC5 significantly reduced the *Hspa9* mRNA expression in the AML12 cell line (Figure 2A). As shown in Figure 2B, the protein level of HSPA9 significantly decreased in response to the lack of TXNDC5 in hepatic cells. Due to our results, TXNDC5 and HSPA9 have protein–protein interactions that stabilize or regulate each other’s expression. The loss of TXNDC5 could disrupt this interaction, leading to the degradation or altered stability of HSPA9, and it also might disrupt a cellular pathway or mechanism that indirectly affects HSPA9 expression. This could be due to a regulatory feedback loop or crosstalk between pathways. In addition, the absence of TXNDC5 might trigger a stress response that influences HSPA9 expression as part of an adaptive cellular mechanism or as an alteration in cellular redox status can impact the gene and protein expression. The mRNA level of *Hspa9* was reduced by 16% and could be the cause of the 20% reduction in the HSPA9 protein level; therefore, there was no evidence or indication of huge degradation due to post-translational modification and the reason for the HSPA9 protein reduction could be the mRNA changes. These could be the reasons that the downregulations were observed in the mRNA and protein levels of HSPA9 in the absence of TXNDC5.

### 2.3. TXNDC5 Absence Reduces PRDX6 Protein Levels

Both TXNDC5 and PRDX6 are involved in redox regulation. TXNDC5, with its thioredoxin domain, participates in thiol–disulfide exchange reactions [21], while PRDX6 directly scavenges ROS [26]. Their functions in redox regulation suggest a potential interplay. Furthermore, TXNDC5 is implicated in protein folding within the ER. If it interacts with PRDX6, this interaction might contribute to the coordination of protein-folding processes and the maintenance of ER proteostasis. The interplay between TXNDC5 and PRDX6 could be part of a broader mechanism to maintain cellular homeostasis, balancing redox status, protein folding, and antioxidant defense [27]. To examine the effect of TXNDC5 deficiency on PRDX6 expression at the mRNA and protein levels. TXNDC5 deletion was incapable of influencing *Prdx6* mRNA expression in the AML12 cell line (Figure 2C). Hence, according to the NCBI blast analysis, which found 86% comparable identities between PRDX6 and PRDX6b, the expression level of *Prdx6b* was also studied, and Figure 2D shows no alteration. Interestingly, the absence of TXNDC5 resulted in a significant reduction in the protein level of PRDX6 in the liver cell line, as shown in Figure 2E. The discordance between the lack of change in the *Prdx6* mRNA expression and the downregulation of the PRDX6 protein levels following TXNDC5 knockout could be attributed to various post-transcriptional and post-translational regulatory mechanisms. Since the *Prdx6* mRNA levels remain constant, changes in the RNA-binding proteins or microRNAs might influence *Prdx6* mRNA translation. On the other hand, it is possible that TXNDC5 plays a role in the folding of PRDX6 by catalyzing thiol–disulfide exchange reactions and helping to maintain the correct redox state of cysteine residues for the formation of its three-dimensional structure and stability, and as a consequence of TXNDC5 deficiency, the degradation rate of the PRDX6 protein might be affected and influence the proper folding of PRDX6 or its interaction with chaperone proteins and be targeted for degradation, leading to a decrease in the PRDX6 protein levels.

### 2.4. Proteasomes Are Not Involved in the Reduction of PRDX6 Protein Induced by the Absence of TXNDC5

The ubiquitin–proteasome system (UPS) represents the main pathway for proteolysis in eukaryotic cells. Given that many cancer cells have increased proteasome activity and a malfunctioning UPS, inhibition of proteasomes in tumor cells results in apoptosis and cell cycle arrest [25]. TXNDC5 knockout could trigger ubiquitination of PRDX6, marking it for proteasomal degradation. This post-translational modification could impact the protein levels without affecting mRNA expression. To address proteasome involvement in PRDX6 protein reduction in the TXNDC5-deficient AML12 cells, cells were incubated in the presence of 5 and 10 µM MG-132 as a proteasome inhibitor for 24 h. The PRDX6 protein levels were assessed via Western blot. As shown in Figure 2F, the WT cells treated with the proteasome inhibitor at both concentrations exhibited PRDX6 bands compared to the control group. This suggests that the proteasome is not involved in the degradation of this protein. This was reinforced in the KO cells, where there was a lack of PRDX6 protein in the presence of proteasome inhibitor.

### 2.5. Lipid Peroxidation Was Decreased in the Absence of TXNDC5

**Figure 3:**
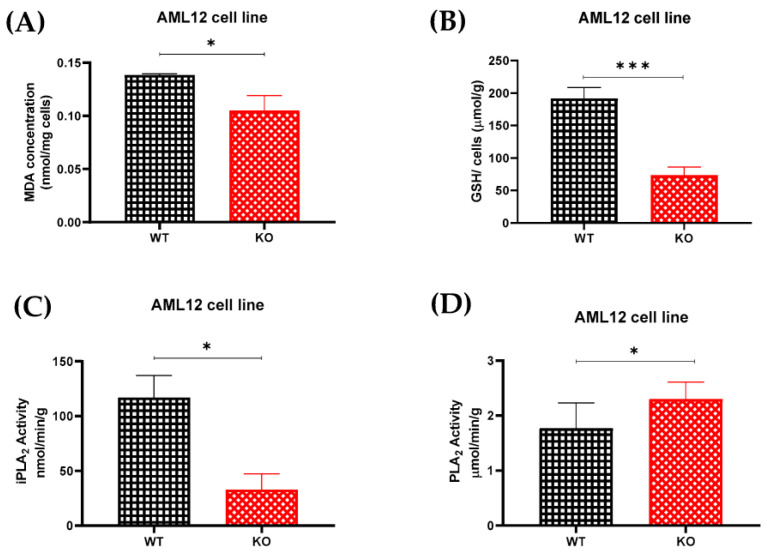
(**A**) Lipid peroxidation analysis. Significant MDA reduction in the KO cells is shown. (**B**–**D**) Assessment of glutathione and iPLA_2_ activities. Considerable decrements of (**B**) GSH and (**C**) iPLA_2_ activity in the KO cells are indicated. (**D**) AML12 KO cells display a remarkable increment in the total PLA_2_. Statistical analysis was carried out according to the Mann–Whitney U test for pairwise comparisons; * *p* < 0.05, *** *p* < 0.001.

PRDX6 is a multi-functional protein with peroxidase activity. Its involvement in redox signaling and lipid peroxidation suggests a potential connection to TXNDC5’s role in redox regulation in the ER [28]. It also plays a crucial role in protecting cells from oxidative stress by scavenging reactive oxygen species and reducing lipid peroxides [29]. An MDA assay was used to compare the levels of lipid peroxidation in the AML12 cell lines (Figure 3A). As shown, the malondialdehyde (MDA) in the AML12 KO cells was significantly decreased compared to the WT cells. TXNDC5’s absence may affect the cellular redox balance through other antioxidant enzymes or pathways that compensate for the loss of this protein, or it may lead to changes in lipid composition due to the phospholipase A_2_ activity of PRDX6, potentially reducing oxidative stress and resulting in a cellular environment less prone to lipid peroxidation and MDA formation. In conclusion, it is possible that TXNDC5 and its interacting protein PRDX6 are involved in the lipid peroxidation processes in AML12 cells.

### 2.6. TXNDC5 Deficiency Decreases Reduced Glutathione and IPLA_2_ Activity

The relationship of TXNDC5 and PRDX6 with glutathione and PLA_2_ involves their interconnected roles in redox regulation, antioxidant defense, and lipid metabolism. PRDX6 interacts directly with glutathione during its peroxidase activity, and it also has phospholipase A_2_ activity, contributing to the remodeling of cellular membranes by releasing fatty acids. This activity is crucial for maintaining lipid homeostasis [30]. As previously described, the elimination of TXNDC5 reduced the protein expression of PRDX6 in the AML12 cell line. To examine the redox status of the cell in relation to the glutathione peroxidase and calcium-independent phospholipase A_2_ (iPLA_2_) activity in the AML12 cell line, GSH, a marker of redox status, and iPLA_2_ activity assays were carried out (Figure 3). As shown in Figure 3B,C, the GSH concentration and iPLA_2_ activity in the AML12 KO cells were significantly decreased compared to the WT cells. Additionally, Figure 3D reveals that the AML12 KO cells had more total PLA_2_ than the WT cells. The knockout of TXNDC5 might trigger compensatory mechanisms to maintain the overall phospholipase activity. When iPLA_2_ is reduced, other PLA_2_ isoforms might be upregulated to compensate for the loss and maintain essential lipid metabolism. As a result, TXNDC5, by interacting with PRDX6, could be a potential mediator in maintaining the overall phospholipase activity.

### 2.7. HSPA9 and PRDX6 Target Genes

**Figure 4:**
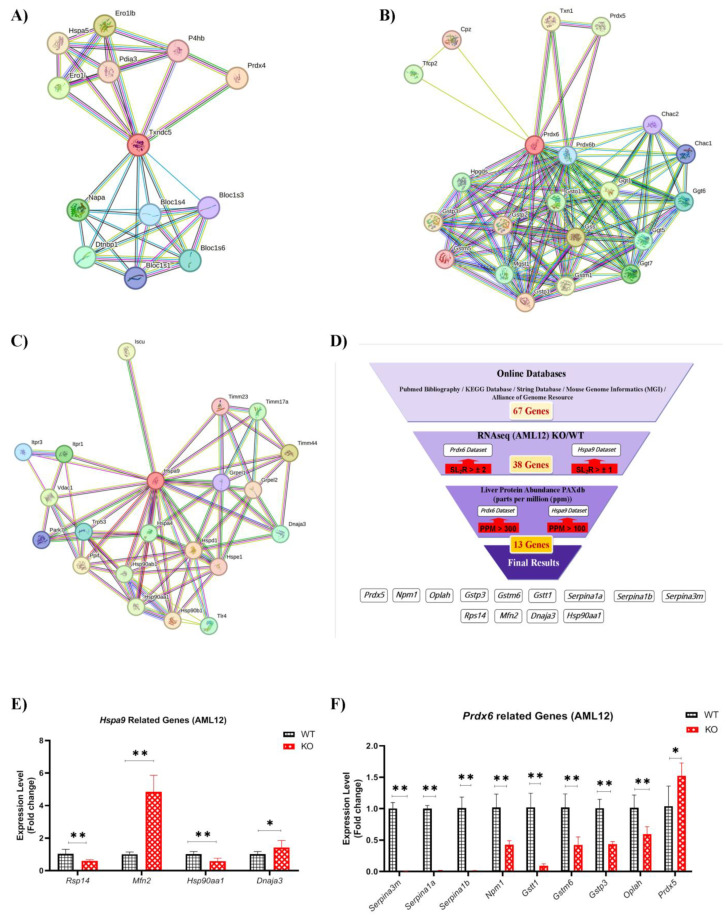
(**A**–**C**) Possible protein–protein interactions of mouse (**A**) TXNDC5, (**B**) PRDX6 and (**C**) HSPA9 generated by the String database. High confidence of 0.7 and not more than 20 interactors are shown. Nodes: network nodes represent proteins; red nodes: query proteins, colored nodes: the first shell of interactors, white nodes: the second shell of interactors. Edges represent protein–protein associations; blue and purple edges: known interactions, green, red and dark-blue edges: predicted interactions, yellow, black and light-blue: others. (**D**) Identification algorithm of genes associated with PRDX6 and HSPA9 based on the PubMed, KEGG, String, Mouse Genome Informatics, and Alliance of Genome Resource databases. Identification was based on mRNA changes according to the RNAseq analysis of the AML12 WT and KO cell lines (SL_2_R > ±2 in the *Prdx6* dataset and SL_2_R > ±1 in the *Hspa9* dataset). The liver protein abundance (parts per million (ppm)) based on the PAX database was considered as a final step (PPM > 300 in the *Prdx6* dataset and PPM > 100 in the *Hspa9* dataset). Expression of (**E**) *Hspa9-* and (**F**) *Prdx6*-associated genes in the AML12 cell line. Data are mean ± SD. Statistical analysis was performed according to the Mann–Whitney U test. * *p* < 0.05, ** *p* < 0.01.

TXNDC5 knockout might influence transcription factors or regulatory elements that control PRDX6 and HSPA9 gene expression. The absence of TXNDC5 may lead to changes in the transcriptional landscape, affecting the expression of downstream genes related to HSPA9 and PRDX6. Using different databases, 67 genes were identified as PRDX6 and HSPA9 targets (Supplementary Table S3). Based on the RNAseq data, 38 genes associated with PRDX6 and HSPA9 showed substantial changes in the transcript levels SL_2_R > ±2 and SL2R > ±1, respectively, between the AML12 WT and KO cell lines (Figure 4D). The PAXdb database was used as a final filter to select 13 genes based on the liver protein abundance (parts per million (PPM)). PPM > 100 and PPM > 300 were also chosen to filter the HSPA9 and PRDX6 datasets, respectively.

RNAseq data obtained from normal and TXNDC5-deficient AML12 cell lines were analyzed, and the represented data were summarized according to transcripts in the liver based on the described databases (Figure 4). This analysis was performed to find the most important genes and modifications based on the absence of TXNDC5 (Supplementary Table S3). The HSBP and MAPK gene families showed the majority of the changes connected to HSPA9, whereas the CHAC, GGT, GSTA, GSTT, PRXL2, RRM and SERPINA gene families indicated the most changes linked to PRDX6. As a result, 13 genes were found to be up- or downregulated in the AML12 cell line (Table 1 and Table 2).

### 2.8. Hepatic Gene Expression

The effect of TXNDC5 on hepatic transcripts linked to *Prdx6* and *Hspa9* was examined using RNA analysis in the AML12 WT and KO cell lines. To confirm the RNAseq data, 13 transcripts were selected from Table 1 and Table 2, including *Dnaja3*, *Hsp90aa1*, *Mfn2*, *Rps14*, *Gstm6*, *Gstp3*, *Gstt1*, *Npm1*, *Oplah*, *Prdx5*, *Serpina1a*, *Serpina1b* and *Serpina3m*. Cell samples were used to set up and validate their RT-qPCR assays. According to the proposed *Hspa9* interactions, TXNDC5 deletion cells exhibit significantly downregulated levels of *Hsp90aa1* and *Rsp14* and significantly upregulated levels of *Dnaja3* and *Mfn2* (Figure 4E). Figure 4F displays the results for all the genes selected to interact with *Prdx6* in the AML12 cell lines. *Prdx5* exhibited a significant increase, whereas *Oplah*, *Gstm6*, *Gstt1* and *Npm1* showed decreased expression in the KO cells. Surprisingly, the SERPINA gene family, including *Serpina1a*, *Serpina1b*, and *Serpina3m*, showed a strong reduction in mRNA levels in the KO cells compared to WT cells.

**Figure 5:**
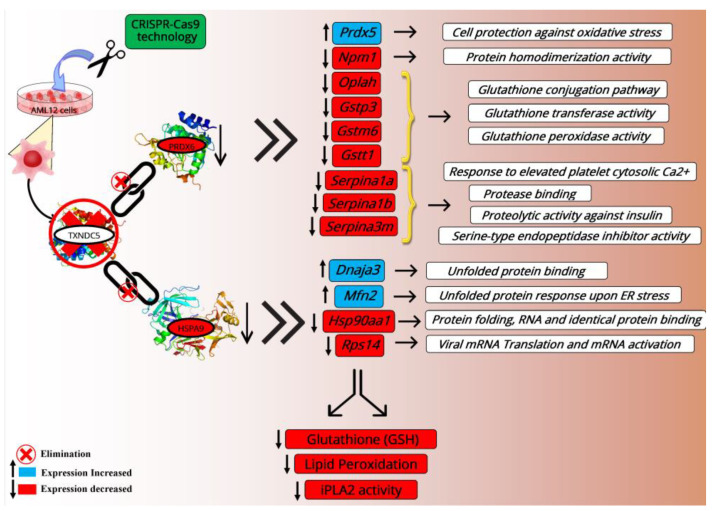
Diagram illustrating the interactions of the TXNDC5 protein with HSPA9 and PRDX6 as well as the impact of TXNDC5 deficiency in AML12 KO cells on different transcriptomes and their functions. Microsoft Publisher Document Version 2010 was used to create this schematic. ↑ Significantly increased, ↓ Significantly decreased. Red circle and cross: Elimination.

A correlation study performed between the RNAseq and RT-qPCR by evaluating the log_2_ fold change values of the transcripts showed a significant agreement (r = 0.91, *p* < 0.0001) in the AML12 cells (Supplementary Figure S3A1), and all the genes were correctly categorized (Supplementary Figure S3A1). However, there were some differences between the signal-log_2_ ratios of the qPCR and RNAseq for *Prdx5* in the AML12 cells. These outcomes reveal that TXNDC5 deficiency and the elimination of its interaction with PRDX6 and HSPA9 can alter the transcriptional landscape, affecting the expression of their downstream genes and multiple cellular processes (Figure 5).

## 3. Discussion

The purpose of this study was to identify novel protein interactions involving TXNDC5 in the liver and the impact that TXNDC5 deficiency on those interactions. We also explored how these interactions affected the activities of iPLA_2_, glutathione (GSH), and lipid peroxidation. Finally, we investigated the mRNA levels of the most significant HSPA9-, PRDX6-, and TXNDC5-related genes. TXNDC5 interacts with a plethora of various proteins and is crucial for numerous cell functions. Histone modification, DNA transcription, mRNA splicing, cell cycle regulation, cell signaling, mobility and transport, metabolism, and protein degradation are among the processes in which it may play a role [1]. Some of these interactions may be important in the progression of certain diseases, including diabetes, neurological diseases, vitiligo, arthritis, and liver cancer [3]. For instance, TXNDC5’s interactions with NENF, PPP1R2, ALDOC, LDH, or PGD may help explain its relevance in diabetes. TXNDC5 dysregulation may influence the cell cycle through cyclin CDK5, histones, or transcription factors like ATF2 or ZNHIT2, which have been implicated as potential carcinogens [38,39]. Moreover, hepatic fat influences TXNDC5, which has been linked to the regulation of ER stress, and may be crucial for the control of apolipoprotein B (APOB) and the development of steatosis [5].

The co-immunoprecipitation (co-IP) and mass spectrometry results identified HSPA9 and PRDX6 as novel TXNDC5-interacting proteins in the AML12 cell line (Figure 1). HSPA9, a member of the heat shock protein 70 family and a ubiquitous molecular chaperone in mammalian cells, has been linked to a variety of biological processes, including protein folding, the assembly of multi-protein complexes, protein trafficking [25], stress responses [40], mitochondrial biogenesis [41], and differentiation [42]. Numerous malignancies, including leukemia, brain cancer, colorectal adenocarcinoma and hepatocellular carcinoma, have been linked to increased expression of *Hspa9* [43,44,45,46]. Metastasis and early tumor recurrence have been associated with *Hspa9* overexpression in liver cancer [47]. Additionally, it has been demonstrated that overexpressing *Hspa9* is adequate to induce breast cancer cells to be more aggressive [46]. These findings suggest that HSPA9 is a promising target for cancer therapy [46]. Also, numerous studies link TXNDC5’s high expression with cancer, and there may be a possible connection between that and HSPA9. According to the results revealed in Figure 2A–C, TXNDC5 inactivation can decrease the expression of HSPA9 mRNA and protein levels, which can induce cell growth arrest and enhance cell apoptosis. These findings are comparable to those of the RNA interference-based selective knockdown of *Hspa9* expression [48].

The reduction in a variety of cellular peroxides is catalyzed by the peroxiredoxin family of proteins, an evolutionarily conserved group of antioxidants that protect cells from oxidative damage [49,50]. The oxidative stress response, cell proliferation, and differentiation are just a few of the biological activities that peroxiredoxins have been linked to collectively [26,27]. H_2_O_2_ and a number of phospholipid peroxides can be reduced by PRDX6, which is cytosolically localized and capable of performing this function [51]. It is highly expressed in several organs, including the liver, lung, and keratinocytes [52,53,54,55,56]. The present study demonstrates that TXNDC5 deficiency does not influence the mRNA level of *Prdx6* in the AML12 cell line, although it induces a remarkable reduction in the protein level (Figure 3C–E). Pathological situations indicate that it may be impacted by *Prdx6* differential expression. According to recent studies, *Prdx6* mRNA levels decrease upon serum deprivation, and keratinocyte growth factor (KGF) is a strong inducer of *Prdx6* expression in both liver cells and keratinocytes. Results also showed that H_2_O_2_ upregulated *Prdx6* in mouse liver cells [29]. Basic biological processes such as cell growth, proliferation, cell cycle, and apoptosis are regulated by the ubiquitin/proteasome system (UPS), and when these activities are dysregulated, malignant transformation results [57]. Since many cancer cells, such as colon and breast cancer, have a defective UPS and elevated proteasome activity, proteasome inhibition in tumor cells has been shown to enhance the accumulation of inhibitors of cyclin-dependent kinases, pro-apoptotic, and tumor suppressor proteins, leading to cell cycle arrest and apoptosis [58,59,60]. To examine whether TXNDC5 could interact with the proteasome and thereby inhibit PRDX6 protein expression, we performed proteasome inhibition via MG-132 in the WT and KO AML12 cell lines; however, the results did not reveal any significant effect on PRDX6 protein expression in the cells, suggesting that the proteasome is not the cellular target of these complexes (Figure 2F).

Recent research has demonstrated that *Prdx6* overexpression can protect lung cells from oxidative stress-induced membrane lipid peroxidation and apoptosis in transfected cells and adenovirus-mediated transfer in mice, whereas *Prdx6* antisense suppression increases oxidative stress susceptibility and cell death [61,62]. However, our results illustrate a significant decrease in lipid peroxidation in the AML12 cell line following the lack of TXNDC5 and reduction in PRDX6 protein (Figure 3A). Alongside lipid peroxidase activity, PRDX6 has been shown to possess glutathione peroxidase and phospholipase A_2_ activities, which are concomitantly increased with *Prdx6* expression. Therefore, this protein plays an important role in membrane phospholipid metabolism [30,63]. The present study, as shown in Figure 3B–D, confirmed that the glutathione (GSH) and iPLA_2_ activities were reduced in the AML12 knockout cells, which is consistent with the study of PRDX6 knockdown in A549, NCI-H460 and H1299 cell lines via siRNA, in which a reduction in the iPLA_2_ and glutathione peroxidase activities was found [30,64].

To assess the impact of *Txndc5* in vivo, RNAseq was previously performed [9], but to determine it in vitro, a next-generation transcriptomic assay was performed. After analysis of RNAseq data from both normal and TXNDC5-deficient AML12 cell lines, the data presented were summarized based on the liver transcripts using the described databases (Figure 4D). In the AML12 cell line, the effect of TXNDC5 on the hepatic transcripts associated with *Hspa9* was examined. From Table 1, the transcripts *Dnaja3*, *Hsp90aa1*, *Mfn2* and *Rps14* were chosen. Our findings show that there is in vitro excessive *Dnaja3* expression. The J-protein *Dnaja3* is in charge of attracting substrates to *Hspa9* and is connected to the non-mitochondrial import functions of *Hspa9* [65]. In two IL-7 responsive B-cell lines (human 697 pre-B and mouse mIL-7R expressing Ba/F3 cells), overexpression of *Dnaja3* inhibits Stat5 phosphorylation and cell proliferation. Reducing the abundance of *Hspa9* may increase the amount of *Dnaja3* in hematopoietic and mouse β-cell lines; however, *Hspa9* and *Dnaja3* have also been established as regulators of *p53* via a similar mechanism [31,66]. However, in liver cells, the absence of TXNDC5 results in a reduction in *Hspa9* and *Dnaja3*. It has been shown that overexpression of *Hsp90* in cancer tissues, particularly in hepatocellular carcinoma, is associated with a poor prognosis and poor therapeutic outcomes [67,68]. *Hsp90*’s prevention of cancer cell proliferation mostly occurs through the degradation of client proteins, including AKT and P53, which in turn causes cancer cells to undergo apoptosis. According to these findings, the combined inhibition of *Hsp90* and *Hspa9* was found to dramatically reduce tumor growth in a liver cancer xenograft model, and the *Hsp90* inhibitor was also found to promote the expression of *Hspa9* [32]. According to the present study, *Hsp90aa1* is downregulated in the absence of TXNDC5 and *Hspa9* is downregulated, which is consistent with previous studies in the liver. Contact points between the endoplasmic reticulum (ER) and mitochondria (referred to as MAMs) in the liver may serve as important nodes for the control of lipid metabolism. However, ER stress was directly associated with altered triglyceride metabolism after *Hspa9* or *Mfn2* overexpression in Huh7 cells, highlighting the importance of the two key partners in establishing MAM integrity and activity [33,69], although *Mfn2* overexpression is present in vitro when *Hspa9* expression is reduced and TXNDC5 is deleted. HSPA9 deletion has been linked to enhanced reactive oxygen species formation in other biological systems. As a consequence, cellular stress is caused by the deletion of RPS14, a gene that is frequently deleted alongside HSPA9 on deletion (5q) as a result of ribosomal insufficiency and P53 activation [31], and haploinsufficiency of deletion genes, such as RPS14 and HSPA9, may play a role in bone marrow failure and inadequate hematopoiesis in hematopoietic cells by activating wild-type TP53 and inducing apoptosis [70]. Nevertheless, TXNDC5 deletion can induce the mRNA activation failure of *Hspa9* and *Rps14* in hepatic cells (Figure 4E).

Accordingly, our results in Figure 4F show that downregulation of TXNDC5 decreased the protein level of PRDX6 in the AML12 cell line, which may alter the expression of several hepatic genes associated with PRDX6 (Table 2). In the AML12 KO cells, *Oplah*, *Gstm6*, *Gstt1*, and *Gstp3*, which play a roles in glutathione metabolism [71], displayed decreased expression. Glutathione synthase deficiency is known to cause oxidative damage to the red blood cell membrane, and another known metabolic defect of the gamma–glutamyl cycle affects an ATP hydrolyzing enzyme (5-oxo-L-prolinase) encoded by the OPLAH gene [72]. Additionally, under oxidative stress, the expression of *Gstm6*, *Gstt1*, and *Gstp3* is decreased in glial cells of the retina, liver, and hepatocytes of rats, respectively [73,74,75]. Our results showed that *Prdx5* was upregulated in the AML12 cells in the absence of TXNDC5, whereas *Prdx5* was downregulated in the PRDX6 knockout HepG2 cells [35]. Recently, it has been shown that *Npm1*, a DNA/RNA chaperone, stimulates *Prdx6* expression and also *Npm1* gene knockdown, suppresses *Prdx6* expression, and on the contrary, an increase in the *Npm1* level can provide an increase in the *Prdx6* level. Furthermore, they are involved in the ROS-P53 pathway and their downregulation can improve the expression level of phosphorylated *p53* and ROS content in HepG2 cells [34,63]. Consequently, our study confirms the *Npm1* decrease in AML12 cells while the PRDX6 protein level is down-expressed. Finally, the mRNA patterns of the predominantly liver-produced *Serpina1a*, *Serpina1b* and *Serpina3m* showed a substantial down-expression in vitro when lacking TXNDC5. Proteins associated with inflammation, such as the SERPINA1 family and TXNDC5, are differentially positively altered in cutaneous squamous cell carcinoma and hepatocytes [36,76]. While the *Serpina3m* transcript levels decreased dramatically in the liver cells, adenomas and carcinomas, they tended to decrease in the non-neoplastic liver cells [37]. Serpin peptidase inhibitor, clade A (alpha-1 antiproteinase, antitrypsin) is the *Serpina3m* gene product that inhibits neutrophil elastase [77]. Due to its tissue-damaging effect, it has been suggested that enhanced neutrophil elastase activity is connected to the generation of carcinogenic responses [37]. These findings imply that the TXNDC5 and PRDX6 complex in the current study may be related to the decreased neutrophil elastase activity caused by *Serpina3m* deficiency. Consistent with these results, perhaps TXNDC5 and its function to fold the proteins and SERPINA family has an unknown interaction through the PRDX6 mediator in the liver.

## 4. Materials and Methods

### 4.1. AML12 Cell Culture

The ATCC collection (Manassas, VA, USA) provided the mouse hepatocyte cell line (AML12), which was cultured in 25 cm^2^ plastic flasks at a density of 5 × 10^5^ cells/cm^2^ (in duplicate) at 37 °C in a humidified atmosphere of 5% CO_2_ in Dulbecco’s modified Eagle’s minimum essential medium (DMEM; Thermo Fisher Scientific, Waltham, MA, USA): F-12-Ham’s medium (GE Healthcare Life Science, South Logan, UT, USA) at a 1:1 ratio supplemented with 10% fetal bovine serum (Thermo Fisher Scientific, Waltham, MA, USA), 1: 500 insulin–transferrin–selenium (Corning, Bedford, MA, USA), 40 ng/mL dexamethasone (Sigma-Aldrich; Merck Millipore, Darmstadt, Germany), 1% non-essential amino acids (Thermo Fisher Scientific, Waltham, MA, USA), 1% amphotericin B (1000 mg/mL; Thermo Fisher Scientific, Waltham, MA, USA), 1% penicillin (1000 U/mL; Thermo Fisher Scientific, Waltham, MA, USA), and 1% streptomycin (1000 mg/mL; Thermo Fisher Scientific, Waltham, MA, USA). Every two days, the culture medium was replaced. After the AML12 cells had achieved 90–100% confluence, the medium was removed and the cells were washed with PBS and trypsinized with 0.25% trypsin (DMEM; Thermo Fisher Scientific, Waltham, MA, USA) and 1 mM EDTA (DMEM; Thermo Fisher Scientific, Waltham, MA, USA). After centrifugation, the cell pellet was stored at −70 °C for RNA and protein extraction.

### 4.2. Creation of a Stable TXNDC5 Knockout AML12 Cell Line

The AML12 cell line was expanded, as previously described [24] Briefly, in order to generate stable clones lacking TXNDC5, the culture medium was removed after one week, the cells were transfected with TXNDC5/ERp46 HDR and TXNDC5 CRISPR/Cas9 KO plasmids (Santa Cruz Biotechnology, Dallas, TX, USA) using Lipofectamine 2000 (Thermo Fisher Scientific, Waltham, MA, USA). The gRNA sequence 5′-TTATCAAGTTCTTCGCTCCG-3′ in the TXNDC5 CRISPR/Cas9 KO plasmid caused a double-strand break (DSB) in the fifth exon of *Txndc5*. The TXNDC5/ERp46 HDR recombined the *Txndc5* gene with a puromycin resistance gene to facilitate the selection of stable knockout AML12 cells [39] AML12 KO cells that were puromycin resistant were selected after repeated puromycin incubations. Western blot confirmed the absence of TXNDC5 (Supplementary Figure S1).

### 4.3. Co-Immunoprecipitation (Co-IP) Assay

Cells were lysed using radioimmunoprecipitation assay (RIPA) lysis buffer for protein extraction, as previously described [78]. Immunoprecipitation was carried out using 1 mg SureBeads ^TM^ Protein A-Magnetic Beads (BioRad, Hercules, CA, USA) in 1.5 mL tubes coupled with 7.5 µg rabbit anti-TXNDC5 (Proteintech, Manchester, UK). Unbound antibody was washed three times with PBS-T (PBS + 0.1% Tween 20). After the sample lysis, the supernatants were incubated with protein A-magnetic beads conjugated to rabbit anti-TXNDC5 overnight at 4 °C. The beads were washed three times with PBS-T. To break the conjugation between the beads and proteins, 20 µL of 20 mM glycine pH 2.0 was added and incubated for 10 min at room temperature. The magnetic beads were captured and the eluent was transferred to a new vial and neutralized with 2 µL (10% eluent volume) of 1 M phosphate buffer pH 7.4. The eluents were analyzed via Western blot analysis.

### 4.4. Protein Identification via LC-MS/MS

Proteomic analysis of the immunoprecipitation from the wild-type and TXNDC5 knockout AML12 cell lines was performed at the Proteomics Unit of the Complutense University of Madrid. Protein samples were boiled for 5 min in loading buffer (6 mM Tris, 2% SDS, 10% glycerol, 0. 5 M beta-mercaptoethanol, traces of bromo-phenol blue) and cooled for 5 min at 4 °C, and they were then loaded on a 4 cm separating gel at 10% acrylamide (acrylamide:bisacrylamide, 19:1) in 1.5 M Tris, pH 8.8, and a 4 cm concentrating gel at 4% acrylamide in 0.5 M Tris, pH 6.5. Electrophoresis was performed in Laemmli buffer at 100 V in a mini-protean system (Bio-Rad, Hercules, CA, USA) until the electrophoretic front (bromophenol blue) had advanced approximately 2 cm in the concentrating gel. The protein bands were visualized by means of staining with colloidal Coomassie (G-250). The bands of the concentrating gel were excised and digested with trypsin. The Coomassie residues and equilibration buffer were removed via two washes with acetonitrile (ACN) alternating with rehydration of the gel with 25 mM ammonium bicarbonate. The disulfide bridges were reduced with 10 mM DTT in 25 mM AMBI at 56 °C for 30 min and blocked with 22.5 mM iodoacetamide in 25 mM AMBI for 15 min in the dark. After removing any traces of the reagents with two ACN washes, the gel was completely dehydrated in a Speed-Vac (Thermo-Savant, Waltham, MA, USA) for 30 min, followed by 0.5 μg of proteomics grade recombinant trypsin (Roche, Madrid, Spain) in 20 μL of 25 mM AMBI and allowed to act overnight at 37 °C. The peptides were collected in the supernatant from the digestion and extraction of the gel with 20 μL acetonitrile. The resulting liquid was blotted dry in a Speed-Vac and reconstituted in 20 µL 2% ACN, 0.1% formic acid. For the peptide separation via reversed-phase chromatography, 10 μL of the digested peptide mixture was injected into the Easy-nLC 1000 nano-HPLC (Thermo), concentrated on a PEPMAP100 C18 NanoViper Trap precolumn (Thermo Fisher Scientific, Waltham, MA, USA), and separated on a 50 cm PEPMAP RSLC C18 column (Thermo) with a gradient of 5% to 40% acetonitrile and 0.1% formic acid in 90 min before loading into the mass spectrometer for analysis. The peptides separated via chromatography were ionized using an electrospray in positive mode and analyzed in a Q Exactive HF (Thermo Fisher Scientific, Waltham, MA, USA) mass spectrometer in DDA (data-dependent acquisition) mode. From each MS scan (between 350 and 1700 Da), the 10 most intense precursors (charge between 2+ and 5+) were selected for their high collision energy dissociation (HCD) fragmentation and the corresponding MSMS spectra were acquired. The data files generated in the shotgun analysis were transferred to Proteome Discoverer 2.4 software (Thermo Fisher Scientific, Waltham, MA, USA), where the PSMs (peptide spectrum matches) of each MSMS spectrum were identified through comparison with the lists of theoretical masses corresponding to the mass of the precursor of origin extracted from the UniProt database (https://www.uniprot.org/) (accessed on 1 November 2020) taxonomically bound to *Mus musculus* and UniProt–Swiss-Prot (all entries) using the Sequest search engine. The identified peptides were assigned to their corresponding proteins.

### 4.5. Western Blot

Proteins were extracted from the AML12 cells, quantified, and transferred to a PVDF membrane, as previously published [24,78]. The membranes were blocked with PBS buffer containing 5% BSA for 1 h at room temperature. After blocking, the membranes were incubated overnight at 4 °C with rabbit primary polyclonal antibodies against mouse TXNDC5 (1:1000, Proteintech, Manchester, UK), PRDX6 (1:1000, Proteintech, Manchester, UK), HSPA9 (1:1000, Proteintech, Manchester, UK), mouse monoclonal anti-HSC70 (1:1000, Proteintech, Manchester, UK) and mouse monoclonal anti-β-ACTIN (1:1000, Sigma, St. Louis, MO, USA). The membranes were washed with PBS solution containing 0.1% Tween 20 before incubation for 1 h at room temperature with conjugated goat anti-rabbit IgG (H&L) DyLight 800 secondary antibody (1:60,000, Thermo-Scientific, Waltham, MA, USA) or goat anti-mouse IgG (H&L) DyLight 680 secondary antibody (1:30,000, Thermo-Scientific, Waltham, MA, USA). The blots were captured using Odyssey^®^ Clx (LI-COR, Bad Hamburg, Germany). The blots were quantified using Image Studio Lite Version 5.2 software (LI-COR Biosciences—GmbH, Bad Homburg, Germany). We expressed the densitometric values normalized to the housekeeping antibodies in arbitrary units.

### 4.6. Bioinformatic Analyses

Data from online bioinformatics databases and a PubMed search were used to confirm and identify the most relevant genes associated with TXNDC5, PRDX6, and HSPA9. In November 2022, 115 and 70 genes were extracted from the PubMed publication date since 2007 using the keywords TXNDC5 and PRDX6, TXNDC5 and HSPA9, respectively. The String database (Figure 4A–C) (https://string-db.org accessed on 20 July 2023) with the criteria of high confidence (0.7) and no more than 20 interactions was used to extract 21 genes per each search, and the KEGG database (https://www.genome.jp/kegg/pathway.html accessed on 20 March 2023) was used to find the 77 and 33 possible genes interacting with the potential biochemical pathways related to PRDX6 and HSPA9 target genes, respectively (Supplementary Figure S2). Then, a total of 67 genes were selected for filtering based on the different databases, including Mouse Genome Informatics (MGI) (http://www.informatics.jax.org accessed on 20 July 2023), PAXdb (https://pax-db.org accessed on 10 September 2023), and Alliance of Genome Resource (https://www.alliancegenome.org accessed on 20 July 2023). The combined information was considered to evaluate the gene and protein expression of their potential target genes in liver.

### 4.7. RNA Extraction

The total cellular RNA was extracted according to the manufacturer’s instructions using a Quick-RNA^TM^ MiniPrep kit (Zymo Research, CA, USA). A spectrophotometer (SPECTROstar^®^, Omega, BMG Labtech, Ortenberg, Germany) was used to measure the amount of RNA based on the absorbance ratio at a wavelength of 260/280 nm. The 28S/18S ratio was greater than 2, which was determined via electrophoresis on a 1% agarose gel, followed by ethidium bromide staining to validate the integrity of the 28S and 18S ribosomal RNAs.

### 4.8. RNAseq Analyses

For the RNA sequencing, the total RNA of the wild-type AML12 (WT) and TXNDC5-knockout AML12 cell lines was prepared. The RNA samples were sequenced at the Beijing Genomics Institute (BGI Genomics, Shenzhen, China). As previously mentioned [79], the following steps were completed: RNA quality testing, library creation, sequencing reads and posterior clean, genome mapping, analysis, identification, and quantification. The average genome mapping rate was 95.95% for the AML12 cells and the full datasets were deposit at GEO (Accession number GSE242049).

### 4.9. Reverse Transcription and Quantitative Real-Time PCR

Quantitative reverse transcriptase PCR assays of these transcripts were optimized for the primer and input cDNA amounts to achieve similar efficiencies. According to the manufacturer’s instructions, 500 ng of extracted total RNA was reverse-transcribed into complementary deoxyribonucleic acid using the PrimeScript RT Reagent Kit (TaKaRa Biotechnology, Kusatsu, Shiga, Japan) in the presence of random and oligo (dT) primers. The primers for each gene were designed using Primer Express (Applied Biosystems, Foster City, CA, USA) (Supplementary Table S1), which were then validated for gene specificity and amplification of cDNA rather than genomic DNA using BLAST analysis (NCBI). Finally, the primers were selected based on the primer efficiency. Quantitative real-time PCR was performed on a StepOnePlus Real-Time PCR System (Applied Biosystems, Foster City, CA, USA) according to the manufacturer’s instructions (SYBR Green PCR Master Mix, Applied Biosystems, Foster City, CA, USA). The relative ratio of the transcript expression level of each gene to the mean values of the control samples was calculated using the comparative 2^−ΔΔCT^ method, normalized to the endogenous control genes *Ppib* and *Tbp*.

### 4.10. Proteasome Inhibition in AML12 WT and KO Cells

The AML12 WT and KO cells, as previously described, were cultured in 25 cm^2^ plastic flasks at a density of 5 × 10^5^ cells/cm^2^ (in duplicate). Afterwards, the medium was removed once the AML12 cells had reached 90–100% confluence, and the cells were washed once with PBS before being incubated in fetal bovine serum-free medium, 5 µM amphotericin B, with 5 µM and 10 µM of MG-132 (Sigma, St Louis, MO, USA) [60] dissolved in DMSO as a proteasome inhibitor. After 24 h of exposure to the proteasome inhibitor, the cells were trypsinized and stored at −20 °C for protein extraction.

### 4.11. Determination of Cellular Lipid Peroxidation via MDA Assay

Malondialdehyde (MDA), an end product of lipid peroxidation, was measured in the AML12 cells using a commercially available MDA assay kit (MAK085, Sigma, Saint Louis, MO, USA).

### 4.12. Total Phospholipase A_2_ (PLA_2_) and Calcium-Independent Phospholipase A_2_ (iPLA_2_) Activity Assays

The PLA_2_ and iPLA_2_ activities were assayed according to the manufacturer’s instructions (Cayman Chemicals, Ann Arbor, MI, USA). Briefly, the cells were homogenized and the supernatants obtained via centrifugation at 10,000× *g* for 15 min at 4 °C. Supernatants containing 50 μg protein in a total volume of 45 μL were added to microplate wells containing 5 μL of assay buffer with (iPLA_2_ activity) or without 10 μM bromoenol lactone (total PLA_2_ activity). The reaction was initiated via the addition of 200 μL of arachidonoyl thio-phosphatidylcholine and was incubated at room temperature for 60 min. By adding 10 µL of 25 mM 5,5’-dithiobis-(2-nitrobenzoic acid), the reaction was stopped, and the absorbance was measured at 405 nm using a microplate reader (SPECTROstar^®^, Omega, BMG Labtech, Ortenberg, Germany). The manufacturer’s recommendations were followed for the calculation of iPLA_2_ activity.

### 4.13. Intracellular Reduced Glutathione (GSH) Concentration Determination

According to the instructions provided by the manufacturer, a kit (38185, Merk KGaA, Darmstadt, Germany) was used to measure the intracellular GSH concentrations in the cells. A kinetic assay was utilized to detect the GSH levels by continuously monitoring the conversion of 5,5′-dithiobis-(2-nitrobenzoic acid) into 5-thio-2-nitrobenzoic acid at 420 nm.

### 4.14. Statistical Analysis

GraphPad Prism 8 (GraphPad, S. Diego, CA, USA) for Windows was used for statistical purposes. The Shapiro–Wilk test and Bartlett’s or Levene’s test were used to assess the homology of variance between the groups and the normal distribution of the data, respectively. Factors meeting both criteria were examined using two-way ANOVA with Dunnett’s multiple comparison test and a two-tailed Student’s *t*-test. If any of the hypotheses failed, statistical analysis was performed using the Mann–Whitney U test. The means and standard deviations of the results are presented. A *p*-value of less than 0.05 was used to indicate statistical significance.

## 5. Conclusions

As a conclusion, our research showed that the HSPA9 and PRDX6 proteins interact with TXNDC5 in AML12 cells. TXNDC5 deficiency reduced the PRDX6 protein levels without altering its transcripts, while the HSPA9 protein and mRNA expression decreased. Lipid peroxidation, reduced-form glutathione (GSH) and iPLA_2_ activity were significantly decreased in the TXNDC5-deficient cells, while the total PLA_2_ was increased. Proteasome suppression via MG-132 in the WT and KO AML12 cell lines revealed no considerable effect on PRDX6 protein expression in the cells, suggesting that the proteasome is not the biological target of the TXNDC5–PRDX6 complex. The effect of TXNDC5 on the hepatic transcripts associated with PRDX6 and HSPA9 revealed that in the AML12 KO cells, *Dnaja3*, *Mfn2*, and *Prdx5* were overexpressed while *Hsp90aa1*, *Rsp14*, *Oplah*, *Gstp3*, *Gstm6*, *Gstt1*, *Serpina1a*, *Serpina1b*, and *Serpina3m* were downregulated (Figure 5).

### Limitations and Future Research

While gene knockdown or knockout experiments can provide valuable insights into the impact of TXNDC5 deficiency on HSPA9 and PRDX6 levels and cell growth, there are some limitations and considerations to be aware of. Cells may activate compensatory mechanisms in response to gene knockout or knockdown, potentially masking the true impact on the HSPA9 and PRDX6 levels and cell growth. Therefore, these results should be validated by using other approaches to knockdown these genes, especially if the study aims to understand the role of TXNDC5 deficiency. The effects of TXNDC5 gene knockout may follow temporal dynamics. Investigating the short- and long-term effects of the temporal dynamics of TXNDC5 deficiency and interaction with PRDX6 and HSPA9 by assessing the early, intermediate and late responses under oxidative stress conditions may be another aspect of future research. This may reveal how cellular responses evolve over time. By addressing these limitations and expanding the research in these suggested directions, future studies can provide a more nuanced understanding of the impact of TXNDC5 deficiency on cellular processes and potentially identify novel therapeutic targets.

## Figures and Tables

**Table 1:** Hepatic transcripts associated with HSPA9.

Gene Symbol	Gene ID (NCBI)	SL_2_R RNAseq (AML12)	Regulation (AML12) KO/WT
*Dnaja3* [31]	83945	1.2331	UP
*Hsp90aa1* [32]	15519	−1.2332	DOWN
*Mfn2* [33]	170731	1.6794	UP
*Rps14* [31]	20044	−1.6093	DOWN

Abbreviations: *Dnaja3*, DnaJ heat shock protein family (Hsp40) member A3; *Hsp90aa1*, heat shock protein 90, alpha (cytosolic), class A member 1; *Mfn2*, mitofusin 2; *Rps14*, ribosomal protein S14.

**Table 2:** Hepatic transcripts linked to PRDX6.

Gene Symbol	Gene ID (NCBI)	SL_2_R RNAseq (AML12)	Regulation (AML12) KO/WT
*Gstm6*	14867	−2.1758	DOWN
*Gstp3*	225884	−2.6291	DOWN
*Gstt1*	14871	−3.3955	DOWN
*Npm1* [34]	18148	−2.3854	DOWN
*Oplah*	75475	−2.5423	DOWN
*Prdx5* [35]	54683	−2.0351	DOWN
*Serpina1a* [36]	20700	−3.8105	DOWN
*Serpina1b* [36]	20701	−2.5978	DOWN
*Serpina3m* [37]	20717	−6.7645	DOWN

Abbreviations: *Gstm6*, glutathione S-transferase, mu 6; *Gstp3*, glutathione S-transferase pi 3; *Gstt1*, glutathione S-transferase, theta 1; *Npm1*, nucleophosmin 1; *Oplah*, 5-oxoprolinase (ATP-hydrolysing); *Prdx5*, peroxiredoxin 5; *Serpina1a*, serine (or cysteine) peptidase inhibitor, clade A, member 1A; *Serpina1b*, serine (or cysteine) peptidase inhibitor, clade A, member 1B; *Serpina3m*, serine (or cysteine) peptidase inhibitor, clade A, member 3M.

## Data Availability

All data are contained within the article and Supplementary Material.

## Supplementary Materials

The following supporting information can be downloaded at https://www.mdpi.com/article/10.3390/ijms242417131/s1.

## References

[1] Horna-Terrón E., Pradilla-Dieste A., Sánchez-de-Diego C., Osada J. (2014). TXNDC5, a newly discovered disulfide isomerase with a key role in cell physiology and pathology. Int. J. Mol. Sci..

[2] Loomba R., Friedman S.L., Shulman G.I. (2021). Mechanisms and disease consequences of nonalcoholic fatty liver disease. Cell.

[3] Ao N., Yang J., Wang X., Du J. (2016). Glucagon-like peptide-1 preserves non-alcoholic fatty liver disease through inhibition of the endoplasmic reticulum stress-associated pathway. Hepatol. Res..

[4] Musso G., Gambino R., Cassader M. (2009). Recent insights into hepatic lipid metabolism in non-alcoholic fatty liver disease (NAFLD). Prog. Lipid Res..

[5] Ramírez-Torres A., Barceló-Batllori S., Martínez-Beamonte R., Navarro M.A., Surra J.C., Arnal C., Guillén N., Acín S., Osada J. (2012). Proteomics and gene expression analyses of squalene-supplemented mice identify microsomal thioredoxin domain-containing protein 5 changes associated with hepatic steatosis. J. Proteom..

[6] Han J., Kaufman R.J. (2016). The role of ER stress in lipid metabolism and lipotoxicity. J. Lipid Res..

[7] Hama Y., Morishita H., Mizushima N. (2022). Regulation of ER-derived membrane dynamics by the DedA domain-containing proteins VMP1 and TMEM41B. EMBO Rep..

[8] Hwang C., Sinskey A.J., Lodish H.F. (1992). Oxidized redox state of glutathione in the endoplasmic reticulum. Science.

[9] Sánchez-Marco J., Martínez-Beamonte R., Diego A.D., Herrero-Continente T., Barranquero C., Arnal C., Surra J., Navarro M.A., Osada J. (2022). Thioredoxin Domain Containing 5 Suppression Elicits Serum Amyloid A-Containing High-Density Lipoproteins. Biomedicines.

[10] Cnop M., Foufelle F., Velloso L.A. (2012). Endoplasmic reticulum stress, obesity and diabetes. Trends Mol. Med..

[11] Fu S., Yang L., Li P., Hofmann O., Dicker L., Hide W., Lin X., Watkins S.M., Ivanov A.R., Hotamisligil G.S. (2011). Aberrant lipid metabolism disrupts calcium homeostasis causing liver endoplasmic reticulum stress in obesity. Nature.

[12] Fu S., Watkins S.M., Hotamisligil G.S. (2012). The role of endoplasmic reticulum in hepatic lipid homeostasis and stress signaling. Cell Metab..

[13] Tan F., Zhu H., He X., Yu N., Zhang X., Xu H., Pei H. (2018). Role of TXNDC5 in tumorigenesis of colorectal cancer cells: In vivo and in vitro evidence. Int. J. Mol. Med..

[14] Alberti A., Karamessinis P., Peroulis M., Kypreou K., Kavvadas P., Pagakis S., Politis P.K., Charonis A. (2009). ERp46 is reduced by high glucose and regulates insulin content in pancreatic β-cells. Am. J. Physiol.-Endocrinol. Metab..

[15] Chawsheen H.A., Jiang H., Ying Q., Ding N., Thapa P., Wei Q. (2019). The redox regulator sulfiredoxin forms a complex with thioredoxin domain–containing 5 protein in response to ER stress in lung cancer cells. J. Biol. Chem..

[16] Hatahet F., Ruddock L.W. (2009). Protein disulfide isomerase: A critical evaluation of its function in disulfide bond formation. Antioxid. Redox Signal..

[17] Benham A.M. (2012). The protein disulfide isomerase family: Key players in health and disease. Antioxid. Redox Signal..

[18] Duivenvoorden W., Hopmans S.N., Austin R.C., Pinthus J.H. (2017). Endoplasmic reticulum protein ERp46 in prostate adenocarcinoma. Oncol. Lett..

[19] Mo R., Peng J., Xiao J., Ma J., Li W., Wang J., Ruan Y., Ma S., Hong Y., Wang C. (2016). High TXNDC5 expression predicts poor prognosis in renal cell carcinoma. Tumor Biol..

[20] Wang L., Song G., Chang X., Tan W., Pan J., Zhu X., Liu Z., Qi M., Yu J., Han B. (2015). The role of TXNDC5 in castration-resistant prostate cancer—Involvement of androgen receptor signaling pathway. Oncogene.

[21] Chawsheen H.A., Ying Q., Jiang H., Wei Q. (2018). A critical role of the thioredoxin domain containing protein 5 (TXNDC5) in redox homeostasis and cancer development. Genes Dis..

[22] Maiorino M., Bosello V., Cozza G., Roveri A., Toppo S., Ursini F. (2011). Glutathione peroxidase-4. Selenium.

[23] Janssen-Heininger Y., Reynaert N.L., van der Vliet A., Anathy V. (2020). Endoplasmic reticulum stress and glutathione therapeutics in chronic lung diseases. Redox Biol..

[24] Bidooki S.H., Alejo T., Sánchez-Marco J., Martínez-Beamonte R., Abuobeid R., Burillo J.C., Lasheras R., Sebastian V., Rodríguez-Yoldi M.J., Arruebo M. (2022). Squalene Loaded Nanoparticles Effectively Protect Hepatic AML12 Cell Lines against Oxidative and Endoplasmic Reticulum Stress in a TXNDC5-Dependent Way. Antioxidants.

[25] Dores-Silva P.R., Cauvi D.M., Kiraly V.T., Borges J.C., De Maio A. (2020). Human HSPA9 (mtHsp70, mortalin) interacts with lipid bilayers containing cardiolipin, a major component of the inner mitochondrial membrane. Biochim. Et Biophys. Acta BBA-Biomembr..

[26] Fujii J., Ikeda Y. (2002). Advances in our understanding of peroxiredoxin, a multifunctional, mammalian redox protein. Redox Rep..

[27] Immenschuh S., Baumgart-Vogt E. (2005). Peroxiredoxins, oxidative stress, and cell proliferation. Antioxid. Redox Signal..

[28] Fujii S., Ushioda R., Nagata K. (2023). Redox states in the endoplasmic reticulum directly regulate the activity of calcium channel, inositol 1, 4, 5-trisphosphate receptors. Proc. Natl. Acad. Sci. USA.

[29] Gallagher B.M., Phelan S.A. (2007). Investigating transcriptional regulation of Prdx6 in mouse liver cells. Free Radic. Biol. Med..

[30] Yun H.-M., Park K.-R., Lee H.P., Lee D.H., Jo M., Shin D.H., Yoon D.-Y., Han S.B., Hong J.T. (2014). PRDX6 promotes lung tumor progression via its GPx and iPLA2 activities. Free Radic. Biol. Med..

[31] Krysiak K., Tibbitts J.F., Shao J., Liu T., Ndonwi M., Walter M.J. (2015). Reduced levels of Hspa9 attenuate Stat5 activation in mouse B cells. Exp. Hematol..

[32] Guo W., Yan L., Yang L., Liu X., E Q., Gao P., Ye X., Liu W., Zuo J. (2014). Targeting GRP75 improves HSP90 inhibitor efficacy by enhancing p53-mediated apoptosis in hepatocellular carcinoma. PLoS ONE.

[33] Bassot A., Prip-Buus C., Alves A., Berdeaux O., Perrier J., Lenoir V., Ji-Cao J., Berger M.-A., Loizon E., Cabaret S. (2021). Loss and gain of function of Grp75 or mitofusin 2 distinctly alter cholesterol metabolism, but all promote triglyceride accumulation in hepatocytes. Biochim. Et Biophys. Acta BBA-Mol. Cell Biol. Lipids.

[34] Chen J., Cao X., Qin X., Liu H., Chen S., Zhong S., Li Y. (2020). Proteomic analysis of the molecular mechanism of curcumin/β-cyclodextrin polymer inclusion complex inhibiting HepG2 cells growth. J. Food Biochem..

[35] López-Grueso M.J., Lagal D.J., García-Jiménez Á.F., Tarradas R.M., Carmona-Hidalgo B., Peinado J., Requejo-Aguilar R., Bárcena J.A., Padilla C.A. (2020). Knockout of PRDX6 induces mitochondrial dysfunction and cell cycle arrest at G2/M in HepG2 hepatocarcinoma cells. Redox Biol..

[36] Azimi A., Kaufman K.L., Ali M., Arthur J., Kossard S., Fernandez-Penas P. (2018). Differential proteomic analysis of actinic keratosis, Bowen’s disease and cutaneous squamous cell carcinoma by label-free LC–MS/MS. J. Dermatol. Sci..

[37] Yafune A., Kawai M., Itahashi M., Kimura M., Nakane F., Mitsumori K., Shibutani M. (2013). Global DNA methylation screening of liver in piperonyl butoxide-treated mice in a two-stage hepatocarcinogenesis model. Toxicol. Lett..

[38] Charlton H.K., Webster J., Kruger S., Simpson F., Richards A.A., Whitehead J.P. (2010). ERp46 binds to AdipoR1, but not AdipoR2, and modulates adiponectin signalling. Biochem. Biophys. Res. Commun..

[39] Heiker J.T., Kosel D., Beck-Sickinger A.G. (2010). Molecular Mechanisms of Signal Transduction via Adiponectin and Adiponectin Receptors. Biol. Chem..

[40] Lu W., Lee N., Kaul S., Lan F., Poon R., Wadhwa R., Luk J. (2011). Mortalin–p53 interaction in cancer cells is stress dependent and constitutes a selective target for cancer therapy. Cell Death Differ..

[41] Ornatsky O., Connor M., Hood D. (1995). Expression of stress proteins and mitochondrial chaperonins in chronically stimulated skeletal muscle. Biochem. J..

[42] Xu J., Xiao H.H., Sartorelli A.C. (1999). Attenuation of the induced differentiation of HL-60 leukemia cells by mitochondrial chaperone HSP70. Oncol. Res..

[43] Pizzatti L., Sá L.A., de Souza J.M., Bisch P.M., Abdelhay E. (2006). Altered protein profile in chronic myeloid leukemia chronic phase identified by a comparative proteomic study. Biochim. Et Biophys. Acta BBA-Proteins Proteom..

[44] Takano S., Wadhwa R., Yoshii Y., Nose T., Kaul S.C., Mitsui Y. (1997). Elevated levels of mortalin expression in human brain tumors. Exp. Cell Res..

[45] Rozenberg P., Kocsis J., Saar M., Prohászka Z., Füst G., Fishelson Z. (2013). Elevated levels of mitochondrial mortalin and cytosolic HSP70 in blood as risk factors in patients with colorectal cancer. Int. J. Cancer.

[46] Wadhwa R., Takano S., Kaur K., Deocaris C.C., Pereira-Smith O.M., Reddel R.R., Kaul S.C. (2006). Upregulation of mortalin/mthsp70/Grp75 contributes to human carcinogenesis. Int. J. Cancer.

[47] Yi X., Luk J.M., Lee N.P., Peng J., Leng X., Guan X.-Y., Lau G.K., Beretta L., Fan S.-T. (2008). Association of mortalin (HSPA9) with liver cancer metastasis and prediction for early tumor recurrence. Mol. Cell. Proteom..

[48] Wadhwa R., Takano S., Taira K., Kaul S.C. (2004). Reduction in mortalin level by its antisense expression causes senescence-like growth arrest in human immortalized cells. J. Gene Med. A Cross-Discip. J. Res. Sci. Gene Transf. Its Clin. Appl..

[49] Hofmann B., Hecht H.-J., Flohé L. (2002). Peroxiredoxins. Biol. Chem..

[50] Rhee S.G., Chae H.Z., Kim K. (2005). Peroxiredoxins: A historical overview and speculative preview of novel mechanisms and emerging concepts in cell signaling. Free Radic. Biol. Med..

[51] Fisher A.B., Dodia C., Manevich Y., Chen J.-W., Feinstein S.I. (1999). Phospholipid hydroperoxides are substrates for non-selenium glutathione peroxidase. J. Biol. Chem..

[52] Sparling N.E., Phelan S.A. (2003). Identification of multiple transcripts for antioxidant protein 2 (Aop2): Differential regulation by oxidative stress and growth factors. Redox Rep..

[53] Wang X., Phelan S.A., Forsman-Semb K., Taylor E.F., Petros C., Brown A., Lerner C.P., Paigen B. (2003). Mice with targeted mutation of peroxiredoxin 6 develop normally but are susceptible to oxidative stress. J. Biol. Chem..

[54] Kim T.-S., Dodia C., Chen X., Hennigan B.B., Jain M., Feinstein S.I., Fisher A.B. (1998). Cloning and expression of rat lung acidic Ca2+-independent PLA2 and its organ distribution. Am. J. Physiol.-Lung Cell. Mol. Physiol..

[55] Frank S., Munz B., Werner S. (1997). The human homologue of a bovine non-selenium glutathione peroxidase is a novel keratinocyte growth factor-regulated gene. Oncogene.

[56] Munz B., FRANK S., HÜBNER G., OLSEN E., WERNER S. (1997). A novel type of glutathione peroxidase: Expression and regulation during wound repair. Biochem. J..

[57] Avram H., Aaron C., Alexander V. (1998). The ubiquitin system. Annu. Rev. Biochem..

[58] Chen L., Madura K. (2005). Increased proteasome activity, ubiquitin-conjugating enzymes, and eEF1A translation factor detected in breast cancer tissue. Cancer Res..

[59] Arlt A., Bauer I., Schafmayer C., Tepel J., Müerköster S.S., Brosch M., Röder C., Kalthoff H., Hampe J., Moyer M. (2009). Increased proteasome subunit protein expression and proteasome activity in colon cancer relate to an enhanced activation of nuclear factor E2-related factor 2 (Nrf2). Oncogene.

[60] Quero J., Cabello S., Fuertes T., Mármol I., Laplaza R., Polo V., Gimeno M.C., Rodriguez-Yoldi M.J., Cerrada E. (2018). Proteasome versus thioredoxin reductase competition as possible biological targets in antitumor mixed thiolate-dithiocarbamate gold (III) complexes. Inorg. Chem..

[61] Manevich Y., Sweitzer T., Pak J.H., Feinstein S.I., Muzykantov V., Fisher A.B. (2002). 1-Cys peroxiredoxin overexpression protects cells against phospholipid peroxidation-mediated membrane damage. Proc. Natl. Acad. Sci. USA.

[62] Pak J.H., Manevich Y., Kim H.S., Feinstein S.I., Fisher A.B. (2002). An antisense oligonucleotide to 1-cys peroxiredoxin causes lipid peroxidation and apoptosis in lung epithelial cells. J. Biol. Chem..

[63] Sharapov M.G., Novoselov V.I., Gudkov S.V. (2019). Radioprotective role of peroxiredoxin 6. Antioxidants.

[64] Lu B., Chen X.-B., Hong Y.-C., Zhu H., He Q.-J., Yang B., Ying M.-D., Cao J. (2019). Identification of PRDX6 as a regulator of ferroptosis. Acta Pharmacol. Sin..

[65] Goswami A.V., Chittoor B., D’Silva P. (2010). Understanding the functional interplay between mammalian mitochondrial Hsp70 chaperone machine components. J. Biol. Chem..

[66] Dhennin-Duthille I., Nyga R., Yahiaoui S., Gouilleux-Gruart V., Régnier A., Lassoued K., Gouilleux F. (2011). The tumor suppressor hTid1 inhibits STAT5b activity via functional interaction. J. Biol. Chem..

[67] Gooljarsingh L.T., Fernandes C., Yan K., Zhang H., Grooms M., Johanson K., Sinnamon R.H., Kirkpatrick R.B., Kerrigan J., Lewis T. (2006). A biochemical rationale for the anticancer effects of Hsp90 inhibitors: Slow, tight binding inhibition by geldanamycin and its analogues. Proc. Natl. Acad. Sci. USA.

[68] Pascale R.M., Simile M.M., Calvisi D.F., Frau M., Muroni M.R., Seddaiu M.A., Daino L., Muntoni M.D., De Miglio M.R., Thorgeirsson S.S. (2005). Role of HSP90, CDC37, and CRM1 as modulators of P16INK4A activity in rat liver carcinogenesis and human liver cancer. Hepatology.

[69] Muñoz J.P., Ivanova S., Sánchez-Wandelmer J., Martínez-Cristóbal P., Noguera E., Sancho A., Díaz-Ramos A., Hernández-Alvarez M.I., Sebastián D., Mauvezin C. (2013). Mfn2 modulates the UPR and mitochondrial function via repression of PERK. EMBO J..

[70] Liu T., Krysiak K., Shirai C.L., Kim S., Shao J., Ndonwi M., Walter M.J. (2017). Knockdown of HSPA9 induces TP53-dependent apoptosis in human hematopoietic progenitor cells. PLoS ONE.

[71] Kanehisa M. (2019). Toward understanding the origin and evolution of cellular organisms. Protein Sci..

[72] Almaghlouth I., Mohamed J., Al-Amoudi M., Al-Ahaidib L., Al-Odaib A., Alkuraya F. (2012). 5-Oxoprolinase deficiency: Report of the first human OPLAH mutation. Clin. Genet..

[73] Wang J., Shanmugam A., Markand S., Zorrilla E., Ganapathy V., Smith S.B. (2015). Sigma 1 receptor regulates the oxidative stress response in primary retinal Müller glial cells via NRF2 signaling and system xc−, the Na+-independent glutamate–cystine exchanger. Free Radic. Biol. Med..

[74] Parl F.F. (2005). Glutathione S-transferase genotypes and cancer risk. Cancer Lett..

[75] Wiegand H., Boesch-Saadatmandi C., Regos I., Treutter D., Wolffram S., Rimbach G. (2009). Effects of quercetin and catechin on hepatic glutathione-S transferase (GST), NAD (P) H quinone oxidoreductase 1 (NQO1), and antioxidant enzyme activity levels in rats. Nutr. Cancer.

[76] Karatas E., Raymond A.-A., Leon C., Dupuy J.-W., Di-Tommaso S., Senant N., Collardeau-Frachon S., Ruiz M., Lachaux A., Saltel F. (2021). Hepatocyte proteomes reveal the role of protein disulfide isomerase 4 in alpha 1-antitrypsin deficiency. JHEP Rep..

[77] Francavilla R., Castellaneta S.P., Hadzic N., Chambers S.M., Portmann B., Tung J., Cheeseman P., Rela M., Heaton N.D., Mieli-Vergani G. (2000). Prognosis of alpha-1-antitrypsin deficiency-related liver disease in the era of paediatric liver transplantion. J. Hepatol..

[78] Herrera-Marcos L.V., Martínez-Beamonte R., Macías-Herranz M., Arnal C., Barranquero C., Puente-Lanzarote J.J., Gascón S., Herrero-Continente T., Gonzalo-Romeo G., Alastrué-Vera V. (2022). Hepatic galectin-3 is associated with lipid droplet area in non-alcoholic steatohepatitis in a new swine model. Sci. Rep..

[79] Abuobeid R., Herrera-Marcos L., Navarro M.A., Arnal C., Martínez-Beamonte R., Surra J., Osada J. (2020). Dietary erythrodiol modifies hepatic transcriptome in mice in a sex and dose-dependent way. Int. J. Mol. Sci..

