# Supplementary material for: Endoplasmic Reticulum Protein TXNDC5 Interacts with PRDX6 and HSPA9 to Regulate Glutathione Metabolism and Lipid Peroxidation in the Hepatic AML12 Cell Line
Source: Int J Mol Sci. 2023 Dec 5;24(24):17131. doi: 10.3390/ijms242417131 (PMC10743020; doi:10.3390/ijms242417131)
Supplement: Supplementary file 1 [file ijms-24-17131-s001.zip › Supplementary Figures.pdf]

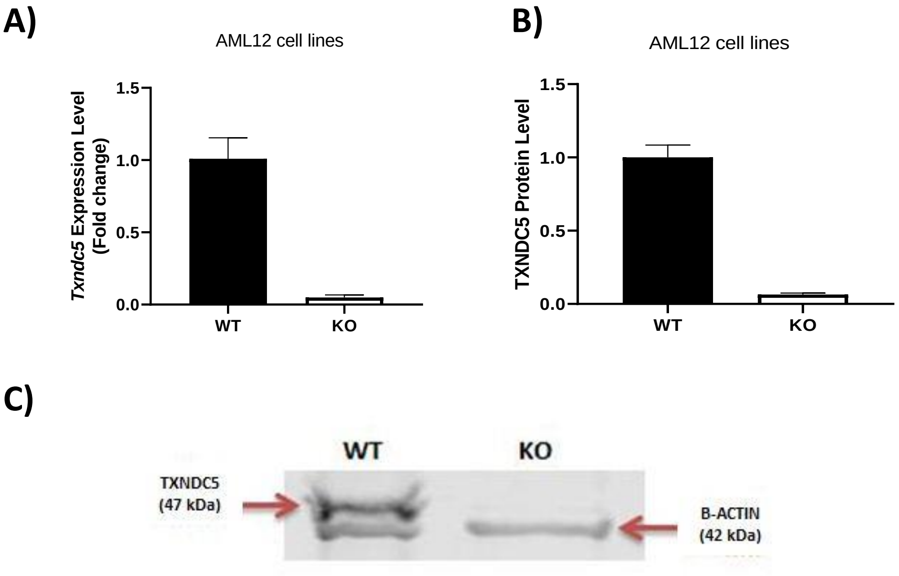

Supplementary Figure S1. Characterization of the AML12 cell lines**. (A)** *Txndc5* mRNA , (**B**) protein levels and (**C**) Western Blot in wildtype (WT) AML12 cells and TXNDC5- knockout (KO) AML12 cells

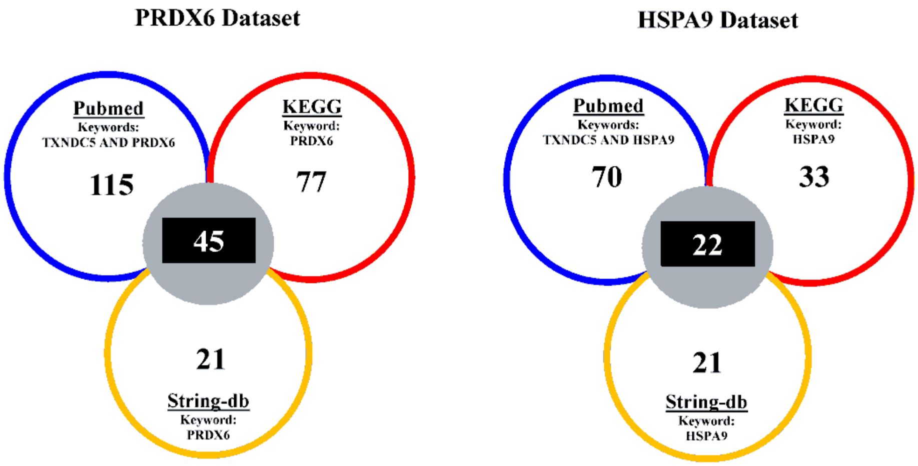

Supplementary Figure S2. Online bioinformatics databases and a PubMed search to confirm and identify the most pertinent genes with TXNDC5, PRDX6, and HSPA9.

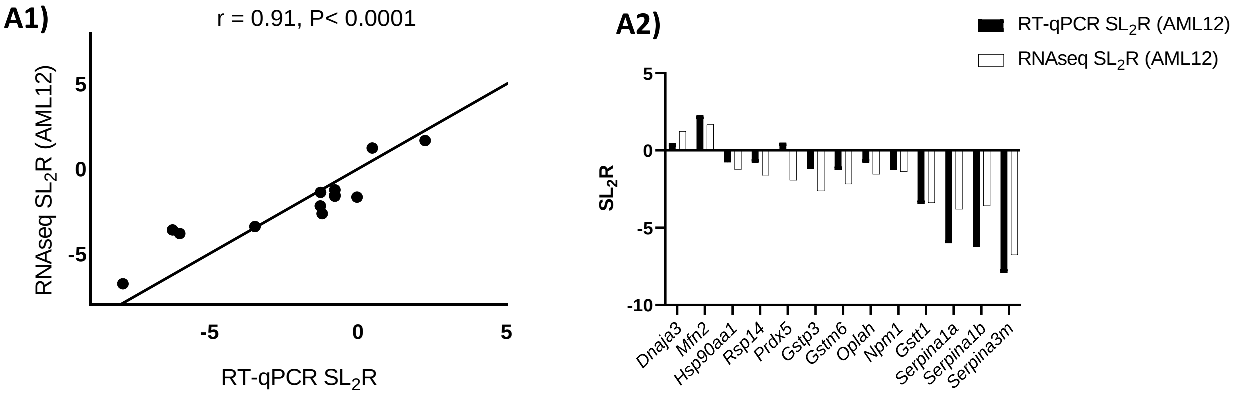

Supplementary Figure S3. Compatibility of RNA analysis techniques. (**A1**) Correlation analysis of 13 selected genes between RNAseq and RT-qPCR normalized to the invariant *Ppib* and *Tbp* genes. The mean values obtained for signal $\log_2$ ratio (SL_2R) from individual analyses in the AML12 cell line. Good agreement between the procedures was observed ($r = 0.91$, $p < 0.0001$). (**A2**) The difference in results of SL_2R expression of both procedures of the 13 selected genes in the AML12 cell line.
