# Supplementary material for: Endoplasmic Reticulum Protein TXNDC5 Interacts with PRDX6 and HSPA9 to Regulate Glutathione Metabolism and Lipid Peroxidation in the Hepatic AML12 Cell Line
Source: Int J Mol Sci. 2023 Dec 5;24(24):17131. doi: 10.3390/ijms242417131 (PMC10743020; doi:10.3390/ijms242417131)
Supplement: Supplementary file 1 [file ijms-24-17131-s001.zip › Supplementary Table S1.pdf]

Supplementary Table S1. Sequences of the real-time PCR primers according to the MIQE guidelines

Gene symbol	Primer sequence, sense/antisense (5'->3')	Amplicon length	Accession	Exon	Concentration	Efficiency
Dnaja3	CCCGGCTTACAGCTTCAGAT<br>TGGTCCTAGACAAGAGCCGT	123	NM_001135112.1, NM_023646.4	12	0.2 µM	99%
Hsp90aa1	CCTGACGGACCCCAGTAAAC<br>TCCACAATGGTCAGGGTTCG	90	NM_010480.5	3	0.2 µM	104%
Mfn2	ATTCACTTCAGAGCAGAGCCA<br>CAGGGTGCCATTCTGAGGAA	379	XM_036163781.1, XM_036163780.1, XM_036163779.1, XM_036163778.1, XM_006538609.5, NM_001355590.1, NM_001355591.1, NM_001285922.1, NM_001285920.1, NM_001285921.1, NM_133201.3, NM_001285923.1	18/19	0.2 µM	96%
Rps14	GGGATGAAGATTGGGCGGAT<br>ACGACCCCCTTTTCTTCGAG	72	NM_020600.4	4/5	0.2 µM	95%
Gstm6	CACGTGGGGCAATGAGTAAG<br>TGGAGGCTTGAGTGAAAGGG	207	XM_036162903.1, XM_006501025.4, NM_001379505.1, NM_001379507.1, NM_001379504.1, XM_006501023.1, NM_001379509.1, NM_008184.4, NM_001379506.1, NM_001379510.1, NM_001379508.1	6	0.2 µM	93%
Gstp3	ACCAGATCTCTTTCGCGGAC<br>ATAGGCGGAGAACAGGGGAA	101	XM_006531725.4, XM_011248620.3, XM_030250873.2, XM_030250874.1, NM_144869.3, NM_001362043.1	2	0.4 µM	87%
Gstt1	CACCTCAGCGATGCGTTTG<br>GGTGAAGCCACCATCCATCA	72	NM_008185.3, NM_001358778.1	2	0.2 µM	94%
Npm1	TTCCCAAAGTGGAAGCCAAGT<br>TCTTGGCAAGTGAACCTGGAC	240	NM_001252260.1, NM_008722.3	10/11	0.2 µM	101%
Oplah	CTTCCATTTTGCCATCGACCG<br>GAGCAGCTTCAGGACACGTA	88	XM_006521509.3, XM_030248806.2, XM_006521508.5, XM_006521507.5, XM_030248807.2, NM_153122.3	2	0.2 µM	98%
Prdx5	AGGCACCTCTTTCTGCGATT<br>GATGCACGGAGCACAGAAC	253	NM_012021.3, NM_001358444.1	1	0.2 µM	99%
Serpina1a	GTCTATGCCCCCTATCCTGC<br>CTATTTGCATGGCTGGAGGAG	369	NM_009243.4, NM_001252569.1	5	0.2 µM	98%
Serpina1b	AGAAGGTTAGTCCAGATCCATATCC<br>GGGCATAGACATAGGAACGGC	246	NM_009244.4	4/5	0.2 µM	94%
Serpina3m	GAAGTTTCTGACCACACGCC<br>CAGGTTGTAGTCAGTAGAGATGGAG	235	XM_006515637.2, NM_009253.2	3/4	0.2 µM	96%
Tbp	GTGAGTTGCTTGCTCTGTGC<br>GCTGCGTTTTTGTGCAGAGT	359	NM_013684.3	8	0.2 µM	104%
Ppib	GGAGATGGCACAGGAGGAA<br>TAGTGCTTCAGCTTGAAGTTCTCAT	71	NM_011149.2	3/4	0.2 µM	99%
Txndc5	CAGGCTTGTCAGATGTCACCAT<br>TAACCTCGTACCGAGTACTTGCTG	82	NM_001289599.1, NM_001289598.1, NM_145367.4	9/10	0.2 µM	92%
Prdx6	CAGCAAACAAGGTAGTGTCAGTG<br>AGGCTTTCTGAGCCCACTTC	218	NM_007453.4, NM_001303408.1, XM_030243163.1	7	0.2 µM	95%
Prdx6B	TATGTCACCAACGATGCCGT<br>CCTTTGACAACAGCAGGCAA	134	NM_177256.5	1	0.2 µM	102%
Hspa9	AAAGTGTGTCAGGGGGAACG<br>TCTGTTGCTCACGACCAGTG	181	NM_010481.2	12/13	0.2 µM	93%

Abbreviations: *Dnaja3,* DnaJ heat shock protein family (Hsp40) member A3; *Hsp90aa1,* heat shock protein 90, alpha (cytosolic), class A member 1; *Mfn2,* mitofusin 2; *Rps14,* ribosomal protein S14; *Gstm6*, glutathione S-transferase, mu 6; *Gstp3*, glutathione S-transferase pi 3; *Gstt1*, glutathione S-transferase, theta 1; *Npm1*, nucleophosmin 1*; Oplah*, 5-oxoprolinase (ATP-hydrolysing); *Prdx5*, peroxiredoxin 5; *Serpina1a*, serine (or cysteine) peptidase inhibitor, clade A, member 1A; *Serpina1b*, serine (or cysteine) preptidase inhibitor, clade A, member 1B*; Serpina3m*,

serine (or cysteine) peptidase inhibitor, clade A, member 3M; *Tbp*, TATA-box binding protein; *Ppib*, peptidylprolyl isomerase B; *Txndc5*, thioredoxin domain containing 5; *Prdx6*, peroxiredoxin 6; *Prdx6B*, peroxiredoxin 6B; *Hspa9*, heat shock protein 9
